# Nomograms Predict Survival Advantages of Gleason Score 3+4 Over 4+3 for Prostate Cancer: A SEER-Based Study

**DOI:** 10.3389/fonc.2019.00646

**Published:** 2019-07-16

**Authors:** Xin Zhu, Xin Gou, Mi Zhou

**Affiliations:** ^1^Department of Urology, The First Affiliated Hospital of Chongqing Medical University, Chongqing, China; ^2^Department of Respiratory and Critical Care Medicine, The First Affiliated Hospital of Chongqing Medical University, Chongqing, China

**Keywords:** prostate cancer, risk factor, gleason score, nomogram, prognosis

## Abstract

**Background:** Different proportions of Gleason pattern 3 and Gleason pattern 4 lead to various prognosis of prostate cancer with Gleason score 7. The objective of this study was to compare the survival outcomes of Gleason score 3+4 and 4+3 based on data from the Surveillance, Epidemiology, and End Results cancer registry database, and to investigate independent prognosis-associated factors and develop nomograms for predicting survival in Gleason score 7 prostate cancer patients.

**Methods:** A retrospective study was conducted on 69,116 cases diagnosed as prostate adenocarcinoma with Gleason score 7 between 2004 and 2009. Prognosis-associated factors were evaluated using univariate and multivariate Cox regression analysis, and a 1:1 ratio paired cohort by propensity score matching with the statistical software IBM SPSS, to evaluate prognostic differences between Gleason score 3+4 and 4+3. The primary cohort was randomly divided into training set (*n* = 48,384) and validation set (*n* = 20,732). Based on the independent factors of prognosis, nomograms for prognosis were established by the training group and validated by the validation group using R version 3.5.0.

**Results:** After propensity score matching, Cox regression analysis showed that Gleason 4+3 had an increased mortality risk both for overall survival (HR: 1.235, 95% CI: 1.179–1.294, *P* < 0.001) and cancer-specific survival (HR: 1.606, 95% CI: 1.468–1.762, *P* < 0.001). Nomograms for overall survival and cancer-specific survival were established with C-index 0.786 and 0.842, respectively. The calibration plot indicated an optimal agreement between the actual observation and nomogram prediction for overall survival and cancer-specific survival probability at 5 or 10 year.

**Conclusions:** Prostate cancer with Gleason score 4+3 had worse overall survival and cancer-specific survival than Gleason score 3+4. Nomograms were formulated to predict 5-year and 10-year OS and CSS in patients with prostate cancer of Gleason score 7.

## Introduction

Prostate cancer remains the most common non-skin male cancer worldwide, with 164,690 estimated new cases and 29,430 estimated deaths in 2018 (1). The Gleason scoring system, the most powerful tool to predict prognosis of patients with prostate cancer (2), was firstly described by Gleason in 1966 (3), and modified in 2005 and more recently in 2014 (4, 5). However, Gleason score 3+4 and 4+3 are often considered the same prognostic group of Gleason score 7, which is one of the major deficiencies of the Gleason scoring system (6). Based on data from Johns Hopkins Hospital, a new grading system was proposed, in which Gleason score 7 is not considered as a single group, but divided into Gleason score 3+4 (prognostic grade group II) and Gleason score 4+3 (prognostic grade group III) (7). Several studies have investigated the prognostic differences between Gleason score 3+4 and Gleason score 4+3; however, the results are inconsistent, and the primary end points emphasize on biochemical free survival (BFS) and progression-free survival (PFS) (7–12). There is still a deficiency in evaluating the survival outcomes of Gleason score 3+4 and 4+3 from perspective of overall survival (OS) and cancer-specific survival (CSS).

Nomograms have been widely proposed as new standards to predict the occurrence and prognosis of various cancer types, including esophageal cancer (13), rectal cancer (14), bladder cancer (15). With regard to prostate cancer, several groups have investigated prognostic nomograms in prostate cancer (16–18). A nomogram consisting of preoperative prostate-specific antigen (PSA), PSA at the time of biochemical recurrence (BCR), the time to BCR, and pathological features, was established and validated to predict the risk of cancer specific mortality after radical prostatectomy (17).

In this study, we aimed to identify independent prognostic factors and specifically, investigate prognosis differences between Gleason score 3+4 and Gleason score 4+3 from the perspective of overall survival and cancer-specific survival using Surveillance, Epidemiology, and End Results (SEER) data. Moreover, the present study also established and validated nomograms to predict OS and CSS in prostate cancer patients with Gleason score 7.

## Materials and Methods

### Data Source

A retrospective analysis of the SEER Program (www.seer.cancer.gov) database was performed under the permission to access the SEER data. The SEER data includes information on cancer incidence in 18 registries across the United States between 1973 and 2015, released April 2018, based on the November 2017 submission. SEER^*^Stat 8.3.5 software was used to extract information from the SEER database (19).

### Study Population

The study population was retrieved from SEER cancer registry with specific inclusion criteria. The inclusion criteria were as follows: (1) Diagnosis of prostate adenocarcinoma (primary sites: C61.9 and ICD-O-3, Hist/behave: 8140/3); (2) diagnosed between 2004 and 2009 with active follow-up to ensure adequate Gleason score records and at least 5-year follow-up data; (3) Gleason scores limited to 3+4 and 4+3; (4) known survival months and specific causes of death; (5) prostate cancer diagnosed as the only primary cancer; (6) known marital status and ethnicity; (7) diagnostic confirmation based on positive histology; (8) known 6th American Joint Committee on Cancer (AJCC) TNM stage, 6th AJCC T status, 6th AJCC N status, and 6th AJCC M status at diagnosis; (9) known PSA values at diagnosis; and (10) surgical conditions limited to no surgeries or radical prostatectomies.

### Study Variables

Study variables extracted from the SEER database included age at diagnosis, race, Gleason score, PSA, AJCC stage, T status, N status, M status, surgical conditions, radiation conditions, and marital status. Patients were divided by age into ≤60 years, 60–70 years, and >70 years. Race was classified as black (African American), white (Caucasian), and others (American Indian/AK Native, Asian/Pacific Islander). Marital status was categorized as married or unmarried (divorced, separated, single, and widowed). PSA was divided into ≤10, 10–20, and >20 ng/ml. AJCC stage was divided by stage II and stage III/IV. T status was categorized as T1/2 and T3/4. N status was described as N0 (negative) and N1 (positive), and M0 indicated negative while M1 indicated positive for M status. Based on surgical and radiotherapy conditions, the therapeutic methods were categorized into four types as follows: no surgery and radiation, only radiation without surgery, only surgery without radiation, and both radiation and surgery.

### Statistical Analysis

Patient demographic characteristics and clinicopathological information were depicted in descriptive method. The chi-squared test was used to assess differences in baseline characteristics between Gleason score 3+4 and 4+3 groups. OS and CSS were the primary end points of the study. OS was defined as time from diagnosis to death due to any cause. CSS was defined as time from diagnosis to the date of cancer specific death. Univariate Cox regression analysis was initially performed to determine factors associated with OS and CSS, then all the related factors were included in the multivariable Cox regression model to assess for prognostic effects on survival. Survival curves were plotted using the Kaplan-Meier method and stratified by prognostic factors identified in the multivariable model. Specifically, a 1:1 ratio paired cohort stratified by Gleason score 3+4 and 4+3 were used to judge the prognostic differences in Gleason score 7 patients.

Then, all the included patients were randomly grouped into training and testing set by a ratio of 7:3. Nomograms for OS and CSS, respectively, were established on the results of multivariate analysis of training group using the package “rms” and “survival” in R version 3.5.0 (http://www.r-project.org). Internal validation of the nomograms was performed using two components. Firstly, the strength of rank correlation between the predicted probability and actual responses was estimated by a concordance index (C-index) in both training and testing set. Secondly, calibration was evaluated by plotting the relationship between actual probability and predicted probabilities with bootstrapping method (1,000 replications) (20).

A 1:1 ratio paired cohort matching by Gleason score was performed by propensity score matching (PSM) using the statistical software IBM SPSS, version 24 (SPSS Inc., Chicago, IL, USA). Survival curves were plotted by Graphpad Prism for windows, version 7.00 (GraphPad, La Jolla, CA, USA). *P* values were two-sided and a threshold of 0.05 was used to determine statistical significance.

## Results

### Patient Characteristics

A total of 69,116 cases diagnosed as prostate adenocarcinoma with either Gleason score 3+4 (50,369) or Gleason score 4+3 (18,747) between 2004 and 2009 were included in the study. Supplementary Figure 1 demonstrated the detailed selection procedure. The clinicopathologic characteristics including age, race, marital status, AJCC stage, T status, N status, M status, PSA level, and therapeutic methods were listed in Supplementary Table 1. Moreover, all the variables stratified by Gleason score 3+4 and 4+3 were compared and demonstrated significant difference (*P* < 0.05). Patients with Gleason score 4+3 were older and had higher PSA values at diagnosis than those with Gleason score 3+4. Moreover, subjects with Gleason score 4+3 were more likely to have advanced AJCC stage, T status, N status, and M status (*P* < 0.05) (Supplementary Table 1). Specifically, the rate of metastasis was 0.8 and 2.3% in patients with Gleason score 3+4 and 4+3 tumors, respectively, suggesting that Gleason score 4+3 is associated with a relatively worse prognosis.

### Independent Prognostic Factors and Impact of Gleason Score on OS

**Figure 1:**
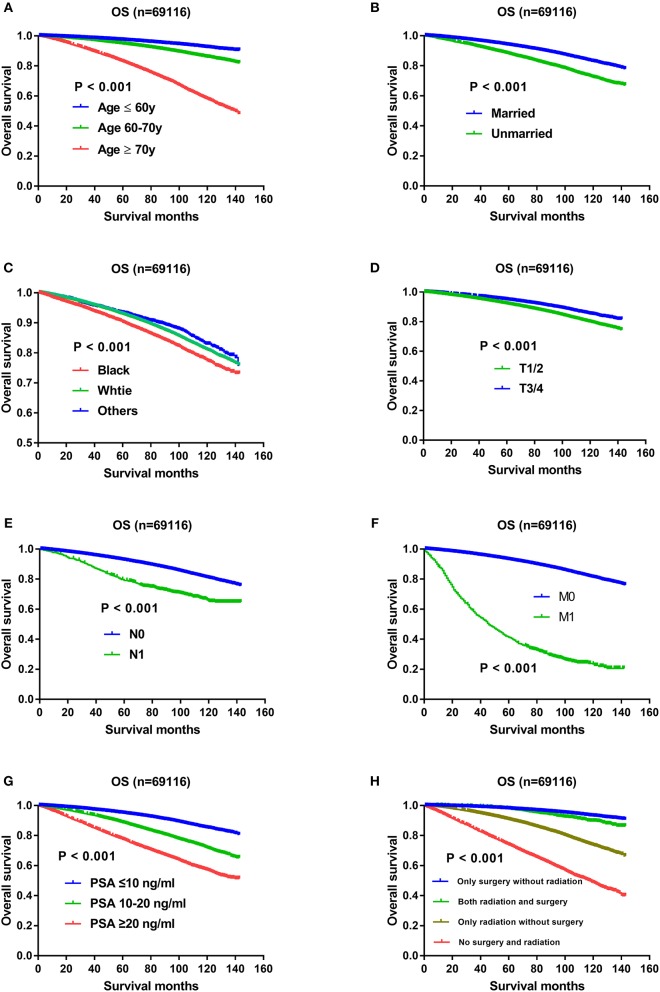
**(A)** Survival curves of OS for age, *P* < 0.001; **(B)** Survival curves of OS for marital status, *P* < 0.001; **(C)** Survival curves of OS for race, *P* < 0.001; **(D)** Survival curves of OS for T status, *P* < 0.001; **(E)** Survival curves of OS for N status, *P* < 0.001; **(F)** Survival curves of OS for M status, *P* < 0.001; **(G)** Survival curves of OS for PSA levels, *P* < 0.001; **(H)** Survival curves of OS for therapeutic methods, *P* < 0.001.

**Figure 2:**
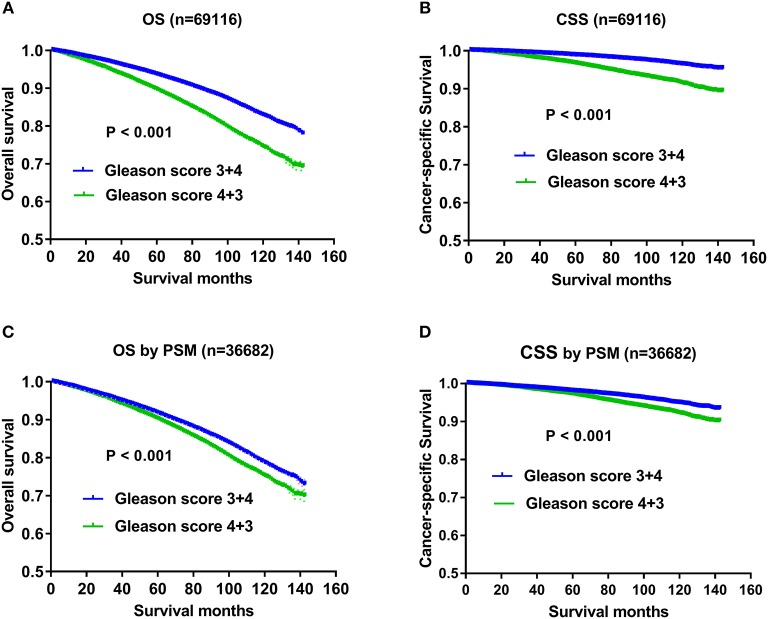
**(A)** Survival curves of OS for Gleason score 3+4 and Gleason 4+3, *P* < 0.001; **(B)** Survival curves of CSS for Gleason3+4 and Gleason 4+3, *P* < 0.001; **(C)** Survival curves of OS for Gleason 3+4 and Gleason 4+3 after PSM in 1:1 ratio, *P* < 0.001; **(D)** Survival curves of CSS for Gleason 3+4 and Gleason 4+3 after PSM in 1:1 ratio, *P* < 0.001.

The results of univariate and multivariate Cox regression analysis on OS were listed in Table 1. Except AJCC stage, all the other variables including Gleason score, age, race, marital status, T status, N status, M status, PSA level, and therapeutic methods were associated with OS significantly in the univariate and multivariate analysis. All the independent prognostic factors except Gleason score were demonstrated in Kaplan–Meier curves, including age (Figure 1A), marital status (Figure 1B), race (Figure 1C), T status (Figure 1D), N status (Figure 1E), *M* status (Figure 1F), PSA levels (Figure 1G), and therapeutic methods (Figure 1H). Specifically, impact of Gleason score 3+4 and Gleason score 4+3 on OS using Kaplan-Meier curves was showed in Figure 2A, which indicated OS advantage of Gleason score 3+4 over Gleason score 4+3 (*P* < 0.001). The Gleason score 3+4 group had better 5-year OS (93.5 vs. 89.60%) and 10-year OS (82.8 vs. 74.4%) than the Gleason score 4+3 group. In the multivariate Cox regression analysis, Gleason score 4+3 was associated with an increased risk for overall mortality compared with Gleason score 3+4 (HR: 1.184, 95% CI: 1.138–1.232, *P* < 0.001) (Table 1).

**Table 1:** Univariate and multivariate Cox analysis of OS.

**Characteristics**		**5-year OS %**	**10-year OS %**	**Univariate analysis**		**Multivariate analysis**	
				**HR [95%CI]**	***P***	**HR [95%CI]**	***P***
Gleason score	3+4 = 7	93.50	82.80	1	<0.001	1	<0.001
	4+3 = 7	89.60	74.40	1.599 [1.538–1.663]		1.184 [1.138–1.232]	
Age at diagnosis	≤60	97.20	92.30	1	<0.001	1	<0.001
	60–70	94.70	85.90	1.898 [1.783–2.021]		1.638 [1.538–1.746]	
	>70	82.90	58	6.705 [6.329–7.103]		3.647 [3.425–3.883]	
Race	Black	90.20	77.40	1	<0.001	1	<0.001
	White	92.80	81	0.797 [0.759–0.837]		0.905 [0.861–0.952]	
	Others	93.20	82.80	0.698 [0.634–0.770]		0.669 [0.607–0.739]	
Marital status	Married	93.80	82.90	1	<0.001	1	<0.001
	Others	87.90	72.30	1.790 [1.720–1.863]		1.461 [1.402–1.522]	
AJCC stage	II	92.60	80.40	1	0.894	NA	NA
	III/IV	91.50	81.00	1.004 [0.953–1.057]		NA	
Stage T	T1/T2	92.10	79.80	1	<0.001	1	<0.001
	T3/T4	94.80	85.20	0.686 [0.645–0.729]		1.329 [1.241–1.423]	
Stage N	N0	92.70	80.80	1	<0.001	1	<0.001
	N1	79.10	65.50	2.159 [1.929–2.417]		1.495 [1.324–1.687]	
Stage M	M0	93.10	81.20	1	<0.001	1	<0.001
	M1	41.30	23.50	9.698 [8.925–10.538]		2.689 [2.452–2.950]	
PSA(ng/ml)	≤ 10	94.90	85.10	1	<0.001	1	<0.001
	10–20	88.50	71.70	2.141 [2.045–2.242]		1.431 [1.365–1.499]	
	>20	77.50	57	3.855 [3.672–4.047]		1.815 [1.720–1.916]	
Treatment	No surgery and radiation	73.90	48.70	1	<0.001	1	<0.001
	Only radiation without surgery	90.70	73.50	0.391 [0.375–0.408]		0.535 [0.512–0.559]	
	Only surgery without radiation	97.80	93.10	0.091 [0.086–0.096]		0.203 [0.190–0.217]	
	Both radiation and surgery	97.90	89.40	0.138 [0.117–0.162]		0.247 [0.208–0.293]	

*NA, Not Available*.

### Independent Prognostic Factors and Impact of Gleason Score on CSS

The results of univariate and multivariate Cox regression analysis on CSS were listed in Table 2. All the variables including Gleason score, age, race, marital status, AJCC stage, T status, N status, M status, PSA level, and therapeutic methods were associated with CSS significantly in the univariate and multivariate Cox analysis (Table 2).

**Table 2:** Univariate and multivariate Cox analysis of CSS.

**Characteristics**		**5-year CSS %**	**10-year CSS %**	**Univariate analysis**		**Multivariate analysis**	
				**HR [95%CI]**	***P***	**HR [95%CI]**	***P***
Gleason score	3+4 = 7	98.70	96.30	1	<0.001	1	<0.001
	4+3 = 7	96.60	91.40	2.538 [2.351–2.740]		1.617 [1.495–1.749]	
Age at diagnosis	≤60	99.00	97.00	1	<0.001	1	
	60–70	98.70	96.10	1.318 [1.182–1.470]		1.153 [1.033–1.288]	0.012
	>70	96.00	90.10	3.602 [3.254–3.987]		1.823 [1.633–2.037]	<0.001
Race	Black	97.50	94.10	1	<0.001	1	
	White	98.20	95	0.812 [0.735–0.897]		1.085 [0.978–1.203]	0.123
	Others	98.50	96.80	0.555 [0.446–0.691]		0.630 [0.505–0.786]	<0.001
Marital status	Married	98.50	95.70	1	<0.001	1	<0.001
	Others	96.80	92.30	1.836 [1.692–1.991]		1.313 [1.207–1.428]	
AJCC stage	II	98.70	96.00	1	<0.001	1	<0.001
	III/IV	94.90	90.00	2.806 [2.589–3.043]		1.763 [1.429–2.175]	
Stage T	T1/T2	98.20	95.20	1	<0.001	1	0.004
	T3/T4	97.70	93.60	1.337 [1.211–1.476]		1.316 [1.090–1.590]	
Stage N	N0	98.30	95.30	1	<0.001	1	<0.001
	N1	84.00	74.00	6.929 [6.045–7.941]		1.649 [1.411–1.928]	
Stage M	M0	98.70	95.70	1	<0.001	1	<0.001
	M1	49.80	34.50	35.753 [32.28–39.60]		4.176 [3.432–5.082]	
PSA (ng/ml)	≤10	99.20	97.00	1	<0.001	1	<0.001
	10–20	97.40	93.40	2.512 [2.268–2.781]		1.588 [1.431–1.762]	
	>20	89.00	79	9.100 [8.347–9.922]		2.936 [2.649–3.253]	
Treatment	No surgery and radiation	90.90	81.80	1	<0.001	1	<0.001
	Only radiation without surgery	98.00	93.80	0.274 [0.252–0.298]		0.490 [0.446–0.537]	
	Only surgery without radiation	99.70	98.50	0.062 [0.055–0.070]		0.133 [0.115–0.154]	
	Both radiation and surgery	98.80	94.30	0.267 [0.214–0.332]		0.308 [0.241–0.394]	

**Figure 3:**
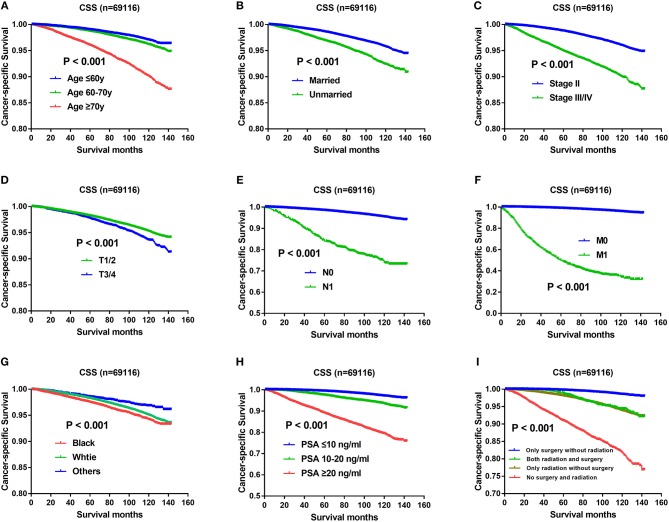
**(A)** Survival curves of CSS for age, *P* < 0.001; **(B)** Survival curves of CSS for marital status, *P* < 0.001; **(C)** Survival curves of CSS for AJCC stage, *P* < 0.001; **(D)** Survival curves of CSS for T status, *P* < 0.001; **(E)** Survival curves of CSS for N status, *P* < 0.001; **(F)** Survival curves of CSS for M status, *P* < 0.001; **(G)** Survival curves of CSS for race, *P* < 0.001; **(H)** Survival curves of CSS for PSA levels, *P* < 0.001; **(I)** Survival curves of CSS for therapeutic methods, *P* < 0.001.

Except Gleason score, independent prognostic factors on CSS were demonstrated in Figure 3, including age (Figure 3A), marital status (Figure 3B), AJCC stage (Figure 3C), T status (Figure 3D), N status (Figure 3E), M status (Figure 3F), race (Figure 3G), PSA levels (Figure 3H), and therapeutic methods (Figure 3I). Specifically, impact of Gleason score 3+4 and Gleason score 4+3 on CSS was showed in Figure 2B, which also indicated CSS advantage of Gleason score 3+4 over Gleason score 4+3 (*P* < 0.001). Gleason score 3+4 had a better 5-year CSS (98.7 vs. 96.6%) and 10-year CSS (96.3 vs. 91.4%) than Gleason score 4+3. In the multivariate analysis, Gleason score 4+3 had a significantly higher cancer-specific mortality risk than Gleason score 3+4 (HR: 1.617, 95% CI: 1.495–1.749) (Table 2).

### Gleason Score on OS and CSS in 1:1 Matched Group by PSM

**Figure 4:**
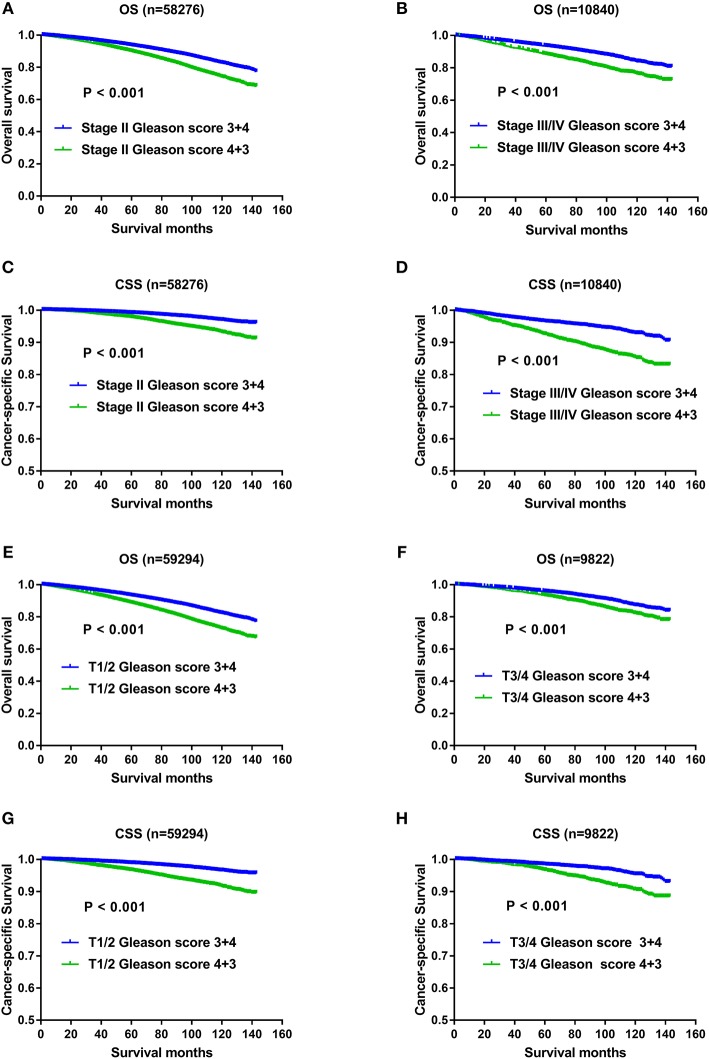
**(A)** Survival curves of OS for Stage II divided by Gleason score, *P* < 0.001; **(B)** Survival curves of OS for Stage III/IV divided by Gleason score, *P* < 0.001; **(C)** Survival curves of CSS for Stage II divided by Gleason score, *P* < 0.001; **(D)** Survival curves of CSS for Stage III/IV divided by Gleason score, *P* < 0.001; **(E)** Survival curves of OS for T1/2 divided by Gleason score, *P* < 0.001; **(F)** Survival curves of OS for T3/4 divided by Gleason score, *P* < 0.001; **(G)** Survival curves of CSS for T1/2 divided by Gleason score, *P* < 0.001; **(H)** Survival curves of CSS for T3/4 divided by Gleason score, *P* < 0.001.

**Figure 5:**
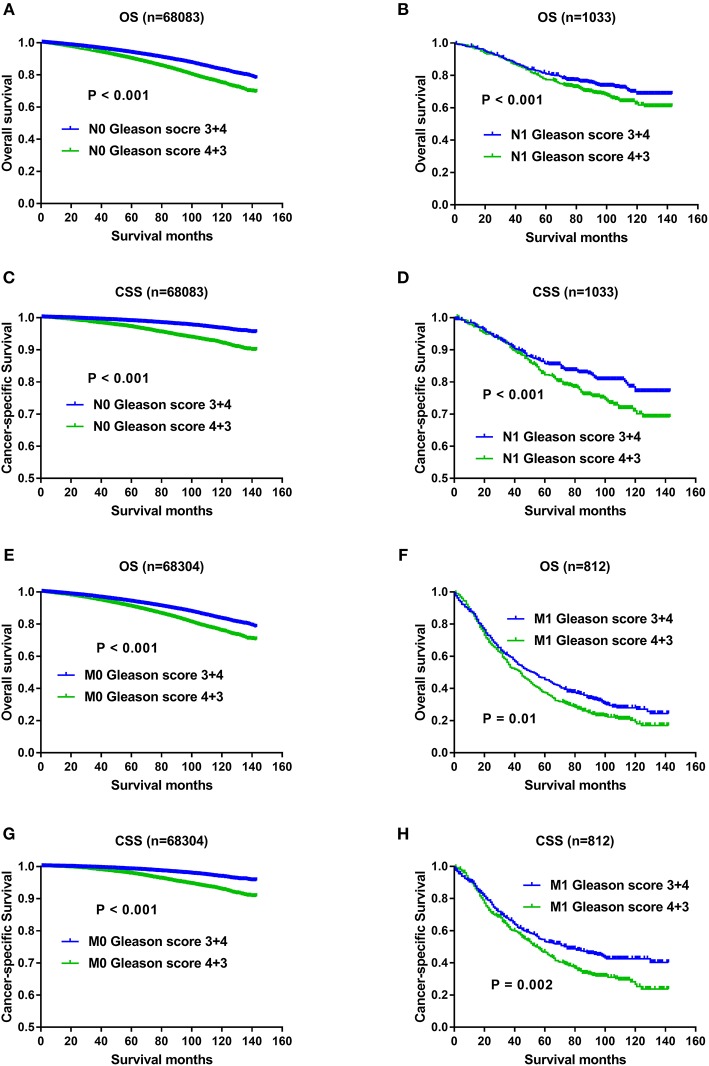
**(A)** Survival curves of OS for N0 divided by Gleason score, *P* < 0.001; **(B)** Survival curves of OS for N1 divided by Gleason score, *P* < 0.001; **(C)** Survival curves of CSS for N0 divided by Gleason score, *P* < 0.001; **(D)** Survival curves of CSS for N1 divided by Gleason score, *P* < 0.001; **(E)** Survival curves of OS for M0 divided by Gleason score, *P* < 0.001; **(F)** Survival curves of OS for M1 divided by Gleason score, *P* = 0.01; **(G)** Survival curves of CSS for M0 divided by Gleason score, *P* < 0.001; **(H)** Survival curves of CSS for M1 divided by Gleason score, *P* = 0.002.

With regard to the significant impact of TNM stage on oncology results, and higher metastatic rate in the Gleason score 4+3 compared with Gleason score 3+4 previously described, it still remained unclear whether the OS and CSS advantage of Gleason score 3+4 was confounded with the effects of TNM status. Therefore, we divided the study population with AJCC stage, T status, N status, M status respectively, and then evaluated whether Gleason score 3+4 still exerted OS and CSS advantage over Gleason score 4+3 after TNM status stratification. As shown in Figure 4, compared with Gleason score 3+4, Gleason score 4+3 still showed OS and CSS disadvantage in Stage II (Figures 4A,C), Stage III/IV (Figures 4B,D), T1/2 (Figures 4E,G),T3/4 (Figures 4F,H), N0 (Figures 5A,C), N1 (Figures 5B,D), M0 (Figures 5E,G), and M1 (Figures 5F,H).

To further evaluate the impact of Gleason score and reduce the confounding effects of all the other prognostic factors on OS and CSS, we matched variables including race, marital status, AJCC stage, T status, N status, M status, PSA level, and therapeutic methods in a 1:1 matched cohort. Finally, 36,682 patients were analyzed including 18,341 patients with Gleason score 3+4 and 18,341 cases with Gleason score 4+3. The demographics and clinicopathological characteristics of the matched cohort are shown in Supplementary Table 2. The confounding factors including age (*P* = 0.922), race (*P* = 0.466), marital status (*P* = 0.98), AJCC stage (*P* = 0.938), T status (*P* = 0.819), N status (*P* = 0.816), M status (*P* = 0.695), PSA level (*P* = 0.944), and therapeutic methods (*P* = 0.996) showed no significant differences between the matched two groups. The survival analysis identified a prognostic advantage for Gleason score 3+4 over Gleason score 4+3 (Figures 2C,D). The 5-year OS and 10-year OS rates were 91.6 and 78.5%, respectively, in the Gleason score 3+4 group, and 89.6 and 74.4%, respectively, in the Gleason 4+3 group. The 5-year CSS and 10-year CSS rates were 97.9 and 94.8%, respectively, in the Gleason score 3+4 group, and 96.6 and 91.4%, respectively, in the Gleason 4+3 group. Multivariate Cox regression analysis showed that Gleason 4+3 had an increased mortality risk both for OS (HR: 1.235, 95% CI: 1.179–1.294, *P* < 0.001) and CSS (HR: 1.606, 95% CI: 1.468–1.762, *P* < 0.001) (Supplementary Table 2).

### Prognostic Nomogram for OS

**Figure 6:**
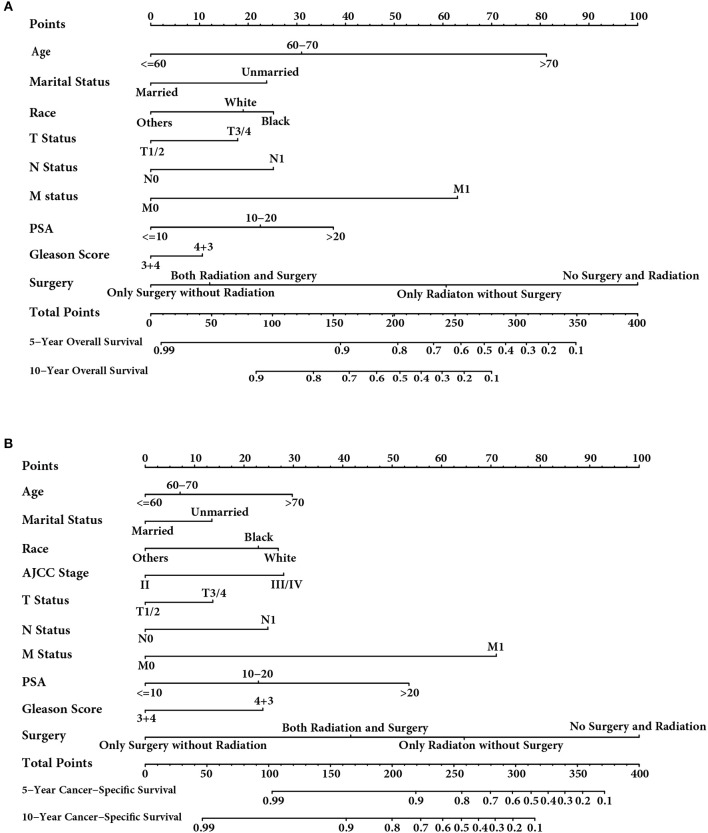
**(A)** Nomogram for OS of Gleason score 7 prostate cancer; **(B)** Nomogram for CSS of Gleason score 7 prostate cancer.

To establish a prognostic nomogram in predicting OS for prostate cancer patients with Gleason score 7, the study population were randomly divided into the training group (48,384) and testing group (20,732) in a 7:3 ratio. The nomogram integrating all the significant independent factors for OS based on the training cohort is shown in Figure 6A. The C-index for nomogram of OS prediction was 0.785 (95% CI, 0.779–0.791), and 0.788 (95% CI, 0.780–0.796) in the training and validation cohort, respectively. The calibration plots indicated an optimal agreement between the actual observation and nomogram prediction for OS probability at 5 and 10 year in the training cohort (Supplementary Figures 2A,C) and testing cohort (Supplementary Figures 2B,D).

### Prognostic Nomogram for CSS

Compared with the nomogram for OS, the nomogram for CSS integrating all the significant independent factors including AJCC stage based on the training cohort is shown in Figure 6B. The C-index for nomogram of CSS prediction was 0.838 (95% CI, 0.828–0.848), and 0.852 (95% CI, 0.838–0.866) in the training and validation cohort, respectively. The calibration plots indicated an excellent accuracy in prediction for CSS probability at 5 and 10 year in the training cohort (Supplementary Figures 3A,C) and testing cohort (Supplementary Figures 3B,D).

## Discussion

The Gleason grading system was used globally in the 1960s to predict the prognosis of males diagnosed with prostate carcinoma (3). It is determined by the sum of the most prevalent pattern and the secondary prevalent pattern in each specimen, resulting in a robust predictor of postoperative progression and survival. Because of the different proportion of Gleason pattern 3 and Gleason pattern 4 leading to various prognosis, Gleason score 7 is categorized as Gleason 3+4 and Gleason 4+3. The dominant pattern in Gleason 7 tumors (score 3+4 vs. 4+3) provides significant prognostic information (7).

Previous studies confirmed the significant difference in biochemical recurrence-free survival (BRFS) following radical prostatectomy between Gleason score 3+4 and 4+3^6−11^. In a study of 263 men with pathological Gleason 7 tumor after radical prostatectomy and a median follow-up of 6.7 years, patients with Gleason score 4+3 were more likely to have seminal vesicle involvement, a higher pathological stage, extraprostatic extension, and higher median preoperative PSA, whereas the score was not independently associated with PFS (11). Sakr et al. found that patients with Gleason score 4+3 had a significantly higher incidence of biochemical recurrence than those with Gleason score 3+4 in the subset of patients with organ-confined prostate cancer (10). Chan et al. investigated 570 cases of Gleason score 7 prostate cancer without lymph node metastasis, seminal vesicle invasion, or tertiary Gleason pattern, and found that a Gleason score of 4+3 was predictive of metastatic disease compared with a Gleason score of 3+4 (9). Alenda et al. found that primary Gleason pattern 4 is an independent predictor of PSA failure based on a single-center cohort of 1,248 patients with Gleason 7 tumors (21). Miyake et al. evaluated the significance of the primary Gleason pattern in 959 consecutive Japanese male patients with Gleason score 7 prostate cancer treated with radical prostatectomy and showed that primary Gleason pattern 4 is significantly associated with the biochemical outcome (12).

Regarding prostate cancer associated mortality, Stark et al. reported that Gleason score 4+3 is associated with a 3-fold increase in lethal prostate cancer compared with Gleason score 3+4 (22). In the present study, a large cohort of patients from the SEER database and a long follow-up time were used to investigate the effects of Gleason score 3+4 and 4+3 on the OS and CSS of prostate cancer. Gleason score 4+3 was associated with worse OS and CSS than Gleason score 3+4 in prostate cancer patients in the multivariate Cox regression analysis and in PSM analysis with other confounding factors excluded.

All the confounding factors including Gleason score, age at diagnosis, race, T stage, N stage, M stage, PSA level, marital status, and therapeutic methods were associated with OS and CSS. With respect to age stratified into different levels, it is easy to understand that the elder group was associated with worse OS and CSS regardless of Gleason score. Regarding race or ethnicity, African American was one of the factors significantly affecting prognosis, and the prostate cancer incidence and mortality of African Americans is among the highest in the world (23). In a study consisting of 1,527,602 eligible prostate cancer cases, Caucasian males had higher 1-year, 3-year, and 5-year net survival than African American males (24). In this study, Caucasian and other ethnic groups including American Indian/AK Native, or Asian/Pacific Islander showed an obvious survival advantage over African Americans. The extensive quality review and validation of the SEER PSA during the 2004–2013 period (25) enabled the inclusion of PSA values in the study, and patients were stratified into three levels: ≤10, 10–20, and >20 ng/ml. Consistent with previous reports and the risk grading of the NCCN guidelines (26), the highest level (>20 ng/ml) showed the lowest survival advantage in patients with Gleason score 7 tumors. Marital status, as a pivotal social exterior factor for cancer patents, has been investigated the associations with outcomes of prostate cancer patients previously (27–29). Although marital status does not affect biochemical recurrence-free and metastases-free survival after radical prostatectomy (28), it is reported to be an independent predictor of OS and CSS in men with prostate cancer, which is consistent with our study (27, 29).

Based on the prognostic factors, we established two nomograms to predict the OS and CSS of prostate cancer patients with Gleason score 7 using the data retrieved from the SEER database. The nomogram for CSS consists of age, race, marital status, AJCC stage, T status, N status, M status, PSA level, and therapeutic methods, and the C-index is 0.838 (95% CI, 0.828–0.848), and 0.852 (95% CI, 0.838–0.866) in the training and validation cohort, with an excellent accuracy in prediction for CSS probability at 5 and 10 year. Except AJCC stage, all the others variables were included in the nomogram for OS, and the C-index was 0.785 (95% CI, 0.779–0.791), and 0.788 (95% CI, 0.780–0.796) in the training and validation cohort, with an optimal agreement in prediction for OS probability.

Although nomograms may provide easier ways to predict OS and CSS of prostate cancer patients, there existed a noticeable point in the nomograms that therapeutic methods seemed to be the most important prognostic factors to predict OS and CSS. In this retrospective study, the therapeutic methods were divided into four groups, including no surgery and radiation, radiation only without surgery, surgery only without radiation, and both radiation and surgery, the latter two surgery groups showed OS and CSS advantage than the other two groups without surgeries. The underlying reason is that all cases included in this study were no surgeries or radical prostatectomy. However, the radiotherapy group was not limited to radical radiotherapy, also included some cases for adjuvant or salvage radiotherapy. Moreover, the group without surgery or radiation has higher positive metastatic rate than the other three groups. Due to advanced status and other socio-economic factors, the group without surgery or radiation had the poorest OS and CSS and exerted the most pronounced effect in the nomograms.

To the best of our knowledge, this study was the first population-based investigation using SEER data to evaluate the different prognostic effects of the Gleason score 3+4 and 4+3 on prognosis of prostate cancer patients. Furthermore, it provided evidence-based data on the largest population from SEER regarding the new grading system based on the proportion of Gleason 4 with regard to OS and CSS. Multivariate regression and PSM analyses indicated that Gleason score 3+4 confers a survival benefit regarding both OS and CSS in patients with prostate cancer. Moreover, the present study provided clinicians with nomograms to predict 5-year and 10-year OS or CSS for prostate cancer with Gleason score 7.

The present study had several limitations. Firstly, because of the lack of follow-up data, factors affecting disease progression after surgery or radiation, such as postoperative serum PSA levels >0.2 ng/ml, evidence of local recurrence, or radiological evidence of distant metastases could not be assessed during the follow-up. This limits further investigation regarding factors affecting biochemical recurrence free survival (BFS), Progression Free Survival (PFS), and other important prognostic indicators. Secondly, this was a retrospective study, and the potential for bias exists even after the application of multivariate analysis and PSM. Thirdly, some significant etiological factors were not recorded in SEER such as body mass index, tobacco, and alcohol use, and specific surgical factors such as positive lymph nodes or seminal vesicle invasion, which also play a significant role in prognosis.

## Conclusion

Despite the limitations mentioned above, the present study demonstrated that Gleason score 4+3 was associated with worse OS and CSS than Gleason score 3+4 in prostate cancer patients and provided nomograms to predict 5-year and 10-year OS as well as CSS in prostate cancer patients with Gleason score 7.

## Ethics Statement

Ethics approval and consent was obtained from SEER database.

## Author Contributions

XZ: data collection, data analysis, and manuscript writing. XG: data analysis and project development. MZ: project development, data collection, and manuscript writing.

### Conflict of Interest Statement

The authors declare that the research was conducted in the absence of any commercial or financial relationships that could be construed as a potential conflict of interest.
