# Supplementary material for: Nomograms Predict Survival Advantages of Gleason Score 3+4 Over 4+3 for Prostate Cancer: A SEER-Based Study
Source: Front Oncol. 2019 Jul 16;9:646. doi: 10.3389/fonc.2019.00646 (PMC6646708; doi:10.3389/fonc.2019.00646)
Supplement: Supplementary file 1 [file Data_Sheet_1.PDF]

frontiers

Supplementary Material

Nomograms predict survival advantages of Gleason score 3+4 over 4+3 for prostate cancer: A SEER-based study

Xin Zhu[1], Xin Gou[1], Mi Zhou[2]

[1] Department of Urology, the First Affiliated Hospital of Chongqing Medical University, Chongqing, 400016, China

[2] Department of Respiratory and Critical Care Medicine, the First Affiliated Hospital of Chongqing Medical University, Chongqing, 400016, China

*** Correspondence:**
Mi Zhou
Email: zhoumi1341@126.com

Supplementary Tables:

Supplementary Table 1. Baseline characteristics of the study population

Supplementary Table 1. Baseline characteristics of the study population

Variants		No.(%) of Patients			P value
		Gleason score 3+4（n=50369）	Gleason score 4+3 （n=18747）	Total（n=69116）	
Age（years）	≤ 60	17948（35.6）	5146（27.4）	23094（33.4）	＜0.001
	60-70	20611（40.9）	7559（40.3）	28170（40.8）	
	＞70	11810（23.4）	6042（32.2）	17852（25.8）	
Race	Black	7772 (15.4)	2935 (15.7)	10707 (15.5)	＜0.001
	White	39975 (79.4)	14636 (78.1)	54611 (79.0)	
	Others	2622 (5.2)	1176 (6.3)	3798 (5.5)	
Marital status	Married	39095(77.6)	14368(76.6)	53463(77.4)	0.006
	Others	11274(22.4)	4379(23.4)	15653(22.6)	
TNM stage	II	43643 (86.6)	14633 (78.1)	58276 (84.3)	＜0.001
	III/IV	6726 (13.4)	4114 (21.9)	10840 (15.7)	
Stage T	T1/T2	44159 (87.7)	15135 (80.7)	59294 (85.8)	＜0.001
	T3/T4	6210 (12.3)	3612 (19.3)	9822 (14.2)	
Stage N	N0	49860 (99.0)	18223 (97.2)	68083 (98.5)	＜0.001
	N1	509 (1.0)	524 (2.8)	1033 (1.5)	
Stage M	M0	49987(99.2)	18317(97.7)	68304(98.8)	＜0.001
	M1	382(0.8)	430(2.3)	812(1.2)	
PSA(ng/ml)	≤ 10	39741(78.9)	12660(67.5)	52401(75.8)	＜0.001
	10-20	7226(14.3)	3651(19.5)	10877(15.7)	

	＞20	3402(6.8)	2436(13.0)	5838(8.4)	
Treatment	No surgery and radiation	5439(10.8)	2799(14.9)	8238(11.9)	＜0.001
	Only radiation without surgery	16637(33.0)	7707(41.1)	24344(35.2)	
	Only surgery without radiation	27163(53.9)	7526(40.1)	34689(50.2)	
	Both radiation and surgery	1130(2.2)	715(3.8)	1845(2.7)	

Supplementary Table 2. Characteristics of patients in 1:1 matched group by PSM.

Supplementary Table 2. Characteristics of patients in 1:1 matched group by PSM

Variants		No.(%) of Patients			P value
		Gleason score 3+4 （n=18341）	Gleason score 4+3 (n=18341)	Total （n=36682）	
Age （years）	≤60	5016(27.3)	5019(27.4)	10035(27.4)	0.922
	60-70	7378(40.2)	7409(40.4)	14787(40.3)	
	＞70	5947(32.4)	5913(32.2)	11860(32.3)	
Race	Black	2807(15.3)	2856(15.6)	5663(15.4)	0.466
	White	14377(78.4)	14378(78.4)	28755(78.4)	
	Others	1157(6.3)	1107(6)	2264(6.2)	
Marital status	Married	14110(76.9)	14112(76.9)	28222(76.9)	0.98
	Others	4231(23.1)	4229(23.1)	8460(23.1)	
AJCC stage	II	14613(79.7)	14619(79.7)	29232(79.7)	0.938
	III/IV	3728(20.3)	3722(20.3)	7450(20.3)	
Stage T	T1/T2	14961(81.6)	14978(81.7)	29939(81.6)	0.819
	T3/T4	3380(18.4)	3363(18.3)	6743(18.4)	

Stage N	N0	18006(98.2)	18000(98.1)	36006(98.2)	0.816
	N1	335(1.8)	341(1.9)	676(1.8)	
Stage M	M0	18078(98.6)	18069(98.5)	36147(98.5)	0.695
	M1	263(1.4)	272(1.5)	535(1.5)	
PSA (ng/ml)	≤ 10	12624(68.8)	12594(68.7)	25218(68.7)	0.944
	10-20	3514(19.2)	3532(19.3)	7046(19.2)	
	＞20	2203(12.0)	2215(12.1)	4418(12.0)	
Treatment	No surgery and radiation	2643(14.4)	2655(14.5)	5298(14.4)	0.996
	Only radiation without surgery	7613(41.5)	7608(41.5)	15221(41.5)	
	Only surgery without radiation	7440(40.6)	7428(40.5)	14868(40.5)	
	Both radiation and surgery	645(3.5)	650(3.5)	1295(3.5)	
	5-year OS rate	91.6%	89.6%		
	10-year OS rate	78.5%	74.4%		
	5-year CSS rate	97.9%	96.6%		
	10-year CSS rate	94.8%	91.4%		
	OS HR [95%CI]	1	1.235, [1.179–1.294]		< 0.001
	CSS HR [95%CI]	1	1.606, [1.468-1.762]		< 0.001

Supplementary Figures:

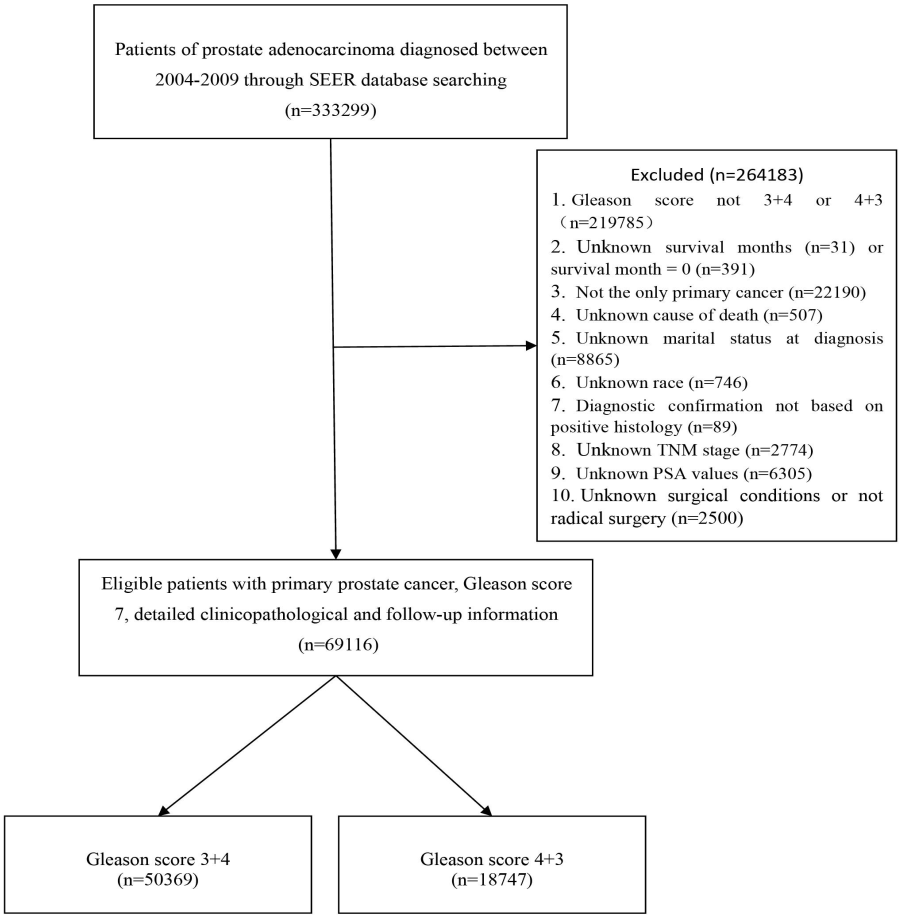

Supplementary Figure 1. The selection procedure of patients of prostate cancer with Gleason score 7 between 2004 and 2009.

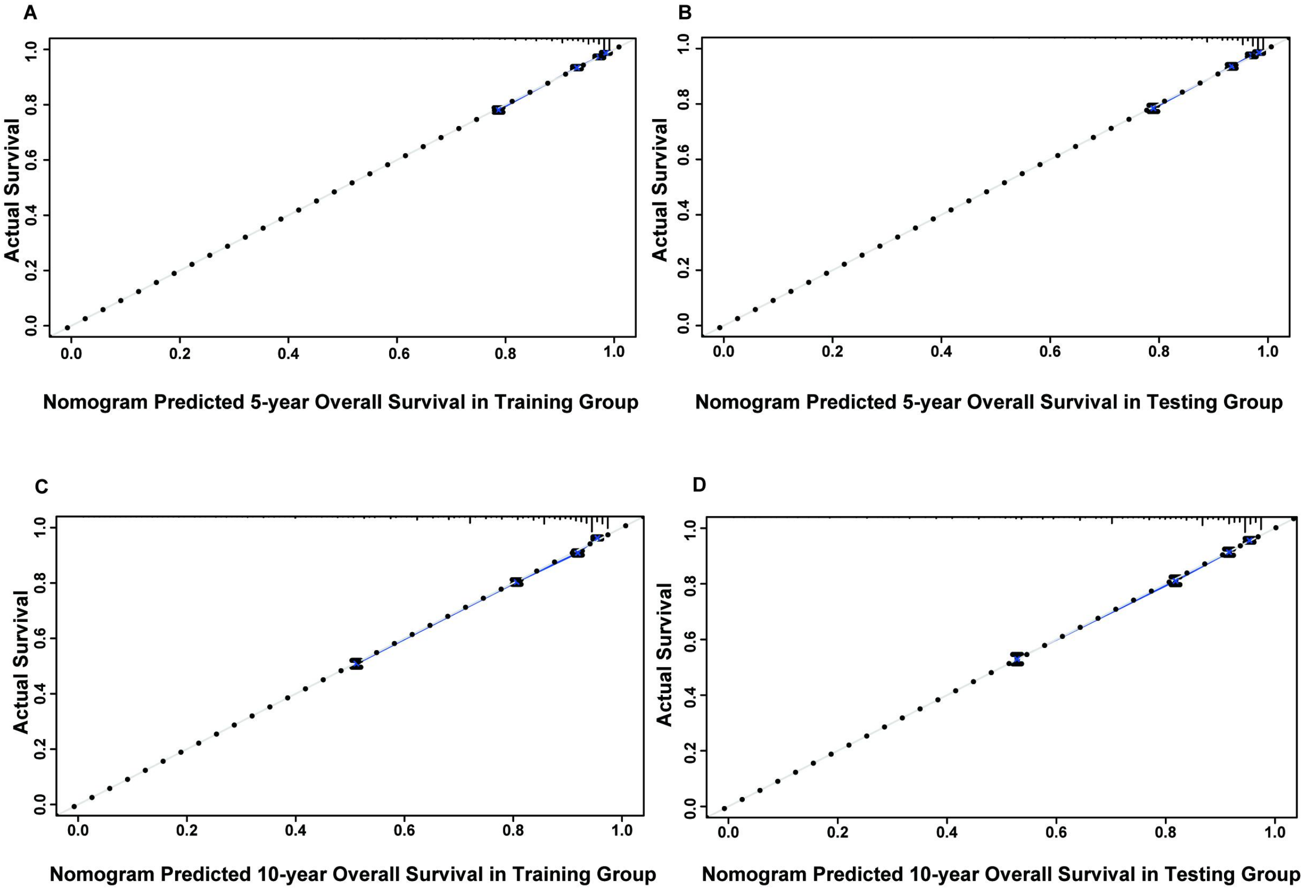

Supplementary Figure 2. 2A.Calibration plot for OS probability at 5 year in the training cohort; 2B.Calibration plot for OS probability at 5 year in the testing cohort; 2C.Calibration plot for OS probability at 10 year in the training cohort; 2D Calibration plot for OS probability at 10 year in the testing cohort.

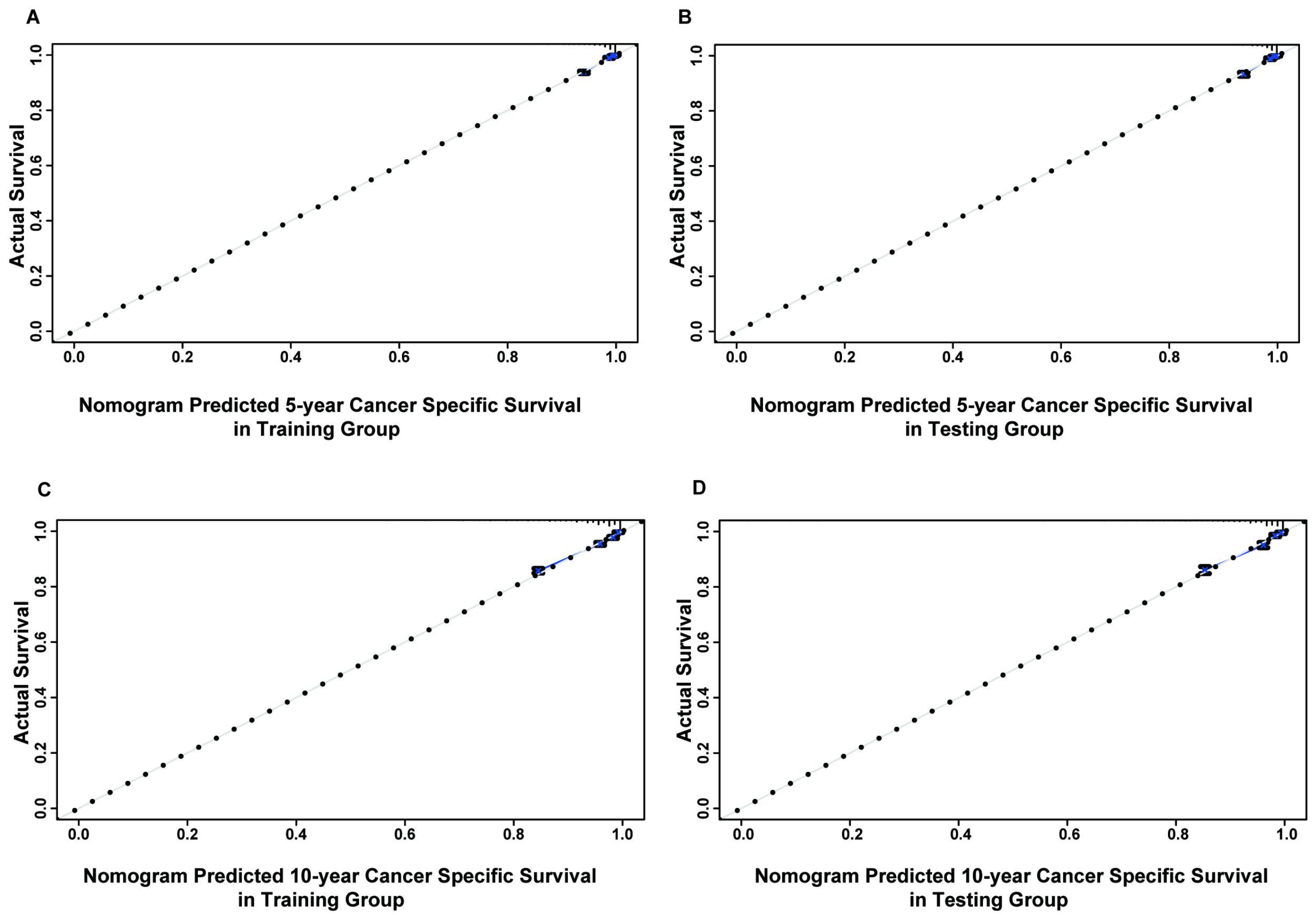

Supplementary Figure 3. 3A.Calibration plot for CSS probability at 5 year in the training cohort; 3B.Calibration plot for CSS probability at 5 year in the testing cohort; 3C.Calibration plot for CSS probability at 10 year in the training cohort; 3D Calibration plot for CSS probability at 10 year in the testing cohort.
